# Risk of hypertension in school-aged children with different parental risk: a longitudinal study from childhood to young adulthood

**DOI:** 10.1186/s12887-021-02807-9

**Published:** 2021-08-17

**Authors:** Parisa Amiri, Marjan Rezaei, Sara Jalali-Farahani, Mehrdad Karimi, Leila Cheraghi, Romina Esbati, Fereidoun Azizi

**Affiliations:** 1 Research Center for Social Determinants of Health, Research Institute for Endocrine Sciences, Shahid Beheshti University of Medical Sciences, Tehran, Iran; 2 Biostatistics Department, Research Institute for Endocrine Sciences, Shahid Beheshti University of Medical Sciences, Tehran, Iran; 3 Endocrine Research Center, Research Institute for Endocrine Sciences, Shahid Beheshti University of Medical Sciences, P.O.Box: 19395-4763, Tehran, Islamic Republic of Iran

**Keywords:** Hypertension, Childhood, Parental risk

## Abstract

**Background:**

Although previous studies have shown the relationship between different parental factors and children’s blood pressure status, there is limited data on the cumulative effect of these factors. Considering parental socio-demographic, behavioral and cardio-metabolic characteristics, the current study aimed to distinguish parental risk clusters and their impact on the incidence of hypertension in school-age children over 13 years.

**Methods:**

Parental characteristics of 1669 children, including age, education, employment, smoking, physical activity, metabolic syndrome (MetS), hypertension (HTN), weight status, and diabetes were considered to categorize parents into low and high-risk clusters. Crude incidence rates (per 10,000 person-years) of HTN in children were assessed in each maternal and paternal cluster. Using Cox proportional hazard model, results on the association between parental risk clusters and HTN incidence in children were reported in five different models.

**Results:**

Mean age of children was 13.96 ± 2.89 years, and 51.2% (*n* = 854) were girls. MetS, HTN, and weight status were the most important factors distinguishing low and high-risk parental clusters, respectively. Crude incidence rates (per 10,000 person-years) of HTN were 86 (95% CI: 71–106) and 38 (95% CI, 29–52) in boys and girls, respectively. Moreover, incidence rates (per 10,000 person-years) of HTN were 50 (95% CI, 40–63) and 80 (95% CI, 64–102) in maternal low and high-risk clusters, respectively. The incidence rates (per 10,000 person-years) of HTN in paternal low and high-risk clusters were 53 (95% CI, 41–70) and 68 (95% CI, 56–84), respectively.

**Conclusion:**

Our findings underscore the prognostic value of maternal characteristics in predicting the incidence of HTN in their offspring. The current results could be valuable in planning related programs to prevent hypertension in similar communities.

## Introduction

Hypertension (HTN) plays a pivotal role in increasing the risk of cardiovascular diseases (CVD) [1]. The World health organization (WHO) has recently reported that 1.13 billion people suffer from HTN worldwide [2]. Pre-hypertension (pre-HTN) and HTN are relatively common in Iran, with approximately one-quarter of Iranian adult populations being hypertensive, the prevalence being higher among elderly populations [3]. In addition, findings of a national survey indicates an alarming rate of high blood pressure in Iranian adolescents, with a high constant trend in both rural and urban areas [4]. Although childhood HTN may not result in HTN-related complications, as usually happens in adults, early stages of complications such as left ventricular hypertrophy (LVH), early or structured atherosclerosis and microalbuminuria do occur in hypertensive children [5]. In addition, data available shows that hypertensive children and adolescents are more likely to suffer from high blood pressure in adulthood, indicating the importance of identifying its related risk factors to prevent this public health crisis in the early years of life [6].

Several hereditary, behavioral, anthropometric, and socio-environmental risk factors underlying HTN in children and adolescents [7, 8]. In this regard, the attributive role of children’s BMI, physical activity, dietary habits, gender and ethnicity in the development of HTN have been investigated previously. Of all these factors, obesity plays a significant role in the incidence of HTN in this age group [5]. In addition, existing data on adolescents shows that blood pressure (BP) is higher in boys, African-Americans, individuals who are less physically active, and those with higher salt consumption [9, 10]. Beyond personal factors, more studies emphasize the importance of the family history of HTN in the elevation of their offspring’s BP [9, 11]. It has been documented that having a history of HTN in both parents, vs one parent magnifies the risk of children’s HTN [12]. Moreover, regarding other parental characteristics, relatively few studies have investigated the association between offspring’s HTN and parental education, smoking and socio-economic status (SES) independently and their findings showed higher rates of adolescents’ HTN and pre-HTN among smoker parents and those with lower education and SES [13–15].

In the Middle-East region, the limited data available regarding parental risk factors of childhood HTN is controversial. While some studies indicate the role of family history of HTN and obesity [16–19], others found no significant contribution of these factors in the development of high BP in children [20, 21]. In Iran, the family history of HTN and obesity has been suggested to be an independent risk factor for children’s HTN [17]. Despite the synergic effects of the aforementioned parental factors on childhood HTN, most previous studies have investigated the association between these factors and childhood HTN separately, resulting in inconsistent findings. Hence, the current research, for the first time, aimed to investigate the association between parental risk clusters and incidence of HTN in offspring in an Eastern-Mediterranean population over a 13-year follow-up.

## Methods

**Fig. 1:**
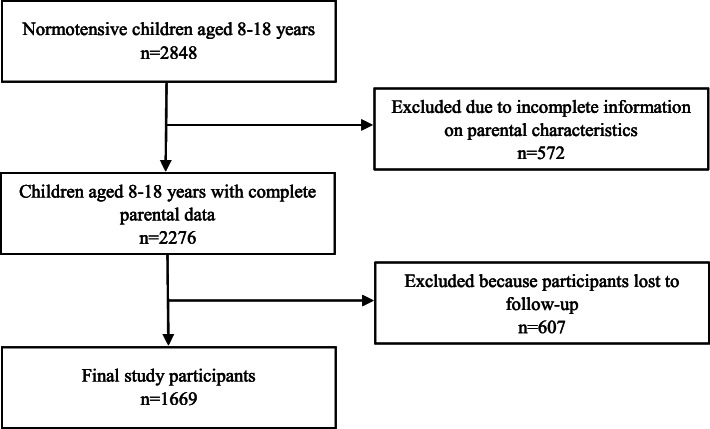
The sampling flowchart

Data for this study was extracted from Tehran Lipid and Glucose Study (TLGS), an ongoing community-based study, initiated in 1999-2001aimed at determining non-communicable diseases (NCD) and their related risk factors in an urban population. The TLGS was designed with two main junctures: Phase I, a cross-sectional study of the prevalence of NCDs and their risk factors, implemented from 1999 to 2001; and Phase II, a prospective follow-up study along with lifestyle interventions and triennial data recollection. Five follow-up re-exams have been carried out from 2002 until 2017 every 3 years. Details on protocol, design, and data collection of the TLGS have been discussed elsewhere [22, 23]. In brief, TLGS participants were selected using a multistage cluster random sampling from three random medical health centers in district 13, located in the eastern part of Tehran. From a total of 15,005 (age, ≥ 3 years) individuals, who had agreed to participate and signed the consent forms, all school-aged children without HTN at baseline (*n* = 2848) and their parents were recruited for the current study, of which 572 children lacked complete parental data, and 607 individuals were lost to follow-up. The remaining 1669 children and their parents were included in the cluster analysis (Fig. 1).

### Study variables

In the current study children’s HTN was considered as the outcome variable. Paternal and maternal risk clusters have been considered as the main predictors of the incidence of HTN in offspring. Parental risk clusters have been defined using socio-demographic (age, education, and employment status), behavioral factors (physical activity and smoking), and cardio-metabolic factors (metabolic syndrome, diabetes, HTN, and weight status). Children’s age, sex, and BMI have been considered as potential confounders.

### Measurements

Measurements were obtained by trained research staff according to the standard protocols [22–24]. Weight was measured with minimum clothing and no shoes using a digital scale. Height was measured while participants were standing with shoulders in normal alignment and without shoes. BMI (kg/m^2^) was calculated to determine weight status in parents and participants aged ≥19 years. Blood pressure was measured twice at 30-s intervals on the right arm at the heart level, while participants were in a relaxed sitting position using a standard mercury sphygmomanometer calibrated by the Iranian Institute of Standards and Industrials Researches. Three different cuffs (pediatrics, regular adult, and large cuff) were available suited for different arm circumferences. Blood pressure was recorded in millimeters of mercury. HTN in adults was defined as systolic blood pressure (SBP) ≥ 140 mmHg, diastolic blood pressure (DBP) ≥ 90 mmHg, or taking antihypertensive medications. For adolescents, HTN was defined as blood pressure equal or greater than the 95th percentile, specific for age, gender and height [25, 26]. Diabetes in parents was defined as fasting blood glucose (FBG) ≥126 mg/dl or 2-h post-glucose challenge ≥200 mg/dl or receiving medications for a definite diagnosis of diabetes. For determining MetS in parents, it was defined according to the joint interim statement, as the presence of any three of the following five risk factors: (1) abdominal obesity with a waist circumference ≥ 95 cm for both genders in Iranian adults [27, 28]; (2) reduced high-density lipoprotein cholesterol (HDL-C) < 50 mg/dl in women, < 40 in men, or receiving medications for reduced HDL-C; (3) elevated triglyceride (TG) levels ≥150 mg/dl or receiving medications for elevated TG; (4) elevated blood pressure (≥130 mmHg SBP or ≥ 85 mmHg DBP) or receiving antihypertensive medications and (5) elevated FBG ≥100 mg/dl or receiving medications for elevated FBG [29]. A trained interviewer used pre-test questionnaires for collecting sociodemographic data, physical activity, and smoking status. Smoking status was categorized into two groups: 1) non-smokers and 2) current smokers (regular or irregular smokers). Information on physical activity was collected with the Iranian version of the Modifiable Activity Questionnaire (MAQ) [30]. The psychometric properties of the Iranian version of the MAQ have been reported previously, and the Iranian version of the questionnaire has been found to have high reliability and moderate validity [31]. In this questionnaire, the number of times and duration of any physical activity is asked. The levels of physical activity have been calculated as MET minutes/week and based on this, the amount of physical activity of individuals divided into three groups including low activity (MET< 600 min/week), moderate (600 ≤ MET< 3000 min/week) and high (MET ≥3000 min / week).

The ethics committee of the Research Institute for Endocrine Sciences (RIES) of Shahid Beheshti University of Medical Sciences approved the current study. Before initiation of the study, all participants provided written informed consent.

### Statistical analysis

The cluster analysis method, a robust statistical method to classify populations, can extract homogenous groups of people used to cluster children. Two-step cluster analysis as a special method which is utilized for both categorical and continues variables was used in this study. Based on Bayesian Information Criteria (BIC), Akaike Information Criteria (AIC) and also considering the interpretability of extracted clusters the optimal number of clusters were stabilized [32]. Two separate cluster analysis were conducted to group children according to maternal and parental risk factors. Based on the parental risk factors, children were classified in four risk groups, including high or low risk maternal clusters and high or low risk paternal clusters. The distribution of categorical factors and the mean of continuous variables were compared between recognized clusters, using the Chi-square test and the independent T-test respectively. Kaplan-Meier curve, Log-rank test and cox proportional hazards model was used to evaluate the parental cluster effects on the incidence of hypertension. In the multiple analysis children’s sex, age and BMI were adjusted as potential confounders. The assumption of proportional hazards in survival cox models was checked by graphically (log minus log graph) and tested by schoenfeld residuals too [33]; *P* < 0.05 was set as significance level and statistical analysis were done by STATA 13 and IBM SPSS 23.

## Results

**Fig. 2:**
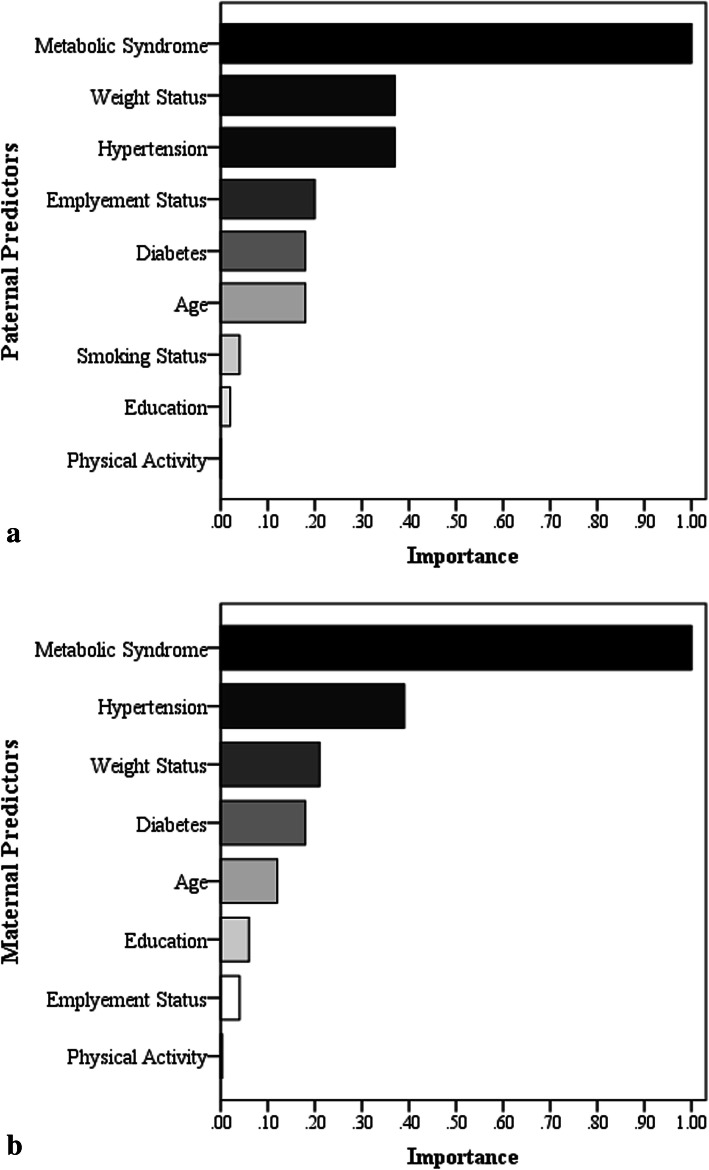
Importance of various factors in the children clustering according to **a** paternal and **b** maternal risk factors associated with hypertension

The mean age of children was 13.96 ± 2.89 years, and 51.2% (*n* = 854) were girls. The median follow-up time was 15.8 years. Parental characteristics, namely, age, education, employment status, MetS status, HTN, diabetes, and body weight status, were used to identify low and high-risk clusters. Figure 2 demonstrates the importance level of mentioned factors in the classification of parental risk clusters. In both parents, the most influential factor was MetS with an importance value of 1, followed by parental HTN and weight status, among other important, significant factors.

Table 1 shows the maternal characteristics of study participants according to their risk clusters. Most children were included in the low-risk maternal cluster (61.1%). As indicated, except for children’s sex and maternal physical activity, all other maternal characteristics were significantly different in the low and high-risk clusters. The mean age of mothers in the high-risk cluster, was significantly higher than in the low-risk cluster. In addition, compared to the low-risk cluster, higher percentages of mothers in the high-risk cluster had primary levels of education, MetS, HTN, diabetes, and obesity. The rate of stay-at-home mothers in the high-risk cluster was also significantly higher than in the low-risk cluster.

**Table 1:** Children’s and mothers’ characteristics across low and high-risk maternal clusters: Tehran Lipid and Glucose Study (TLGS)

		Maternal risk clusters		
	Total *n* = 1669	Low risk *n* = 1019 (61.1)	High risk *n* = 650 (38.9)	***P***-value
**Child’s characteristics**				
**Age (years)**	13.96 ± 2.89	13.63 ± 2.94	14.47 ± 2.73	< 0.001
**BMI (Kg/m**^**2**^)	19.43 ± 3.96	18.94 ± 3.79	20.20 ± 4.10	< 0.001
**Sex**				0.79
Male	815(48.8)	495(48.6)	320(49.2)	
Female	854(51.2)	524(51.4)	330(50.8)	
**Maternal characteristics**				
**Age (years)**	40.27 ± 6.90	38.61 ± 6.16	42.87 ± 718	< 0.001
**Education**				< 0.001
Illiterate/ Primary	616(36.9)	293(28.8)	323(49.7)	
Secondary	972(58.2)	657(64.5)	315(48.5)	
Higher	81(4.9)	69(6.8)	12(1.8)	
**Employment**				< 0.001
Housewife	1521(91.1)	891(87.4)	630(96.9)	
Employed	148(8.9)	128(12.6)	20(3. 1)	
**Metabolic syndrome**				< 0.001
No	1059(63.5)	996(97.7)	63(9.7)	
Yes	610(36.5)	23(2.3)	587(90.3)	
**Hypertension**				< 0.001
No	1368(82)	1009(99)	359(55.2)	
Yes	301(18)	10(1)	291(44.8)	
**Bodyweight status**				< 0.001
Normal	359(21.5)	313(30.7)	46(7.1)	
Overweight	755(45.2)	511(50.1)	244(37.5)	
Obese	555(33.3)	195(19.1)	360(55.4)	
**Diabetes**				< 0.001
No	1494(89.5)	1006(98.7)	488(75.1)	
Yes	175(10.5)	13(1.3)	162(24.9)	
**Physical activity**				0.98
Low	1074(64.3)	655(64.3)	419(64.5)	
Moderate	193(11.6)	117(11.5)	76(11.7)	
High	402(24.1)	247(24.2)	155(23.8)	

Table 2 displays paternal characteristics of study participants by risk clusters. More than half of the children were included in the paternal high-risk cluster (54.5%). Like maternal characteristics, except for children’s sex and paternal physical activity, all other paternal characteristics were significantly different in the low and high-risk clusters. The mean age of fathers was significantly higher in the high-risk cluster compared to the low-risk cluster. Moreover, higher percentages of fathers had a primary level of education, MetS, HTN, diabetes, and obesity in the high-risk cluster compared to the low-risk one. However, the percentage of fathers’ smoking was significantly higher in the low-risk cluster than in the high-risk cluster.

**Table 2:** Children’s and fathers’ characteristics across low and high-risk paternal clusters: Tehran Lipid and Glucose Study (TLGS)

		Paternal risk clusters		
	Total *n* = 1669	Low risk *n* = 760 (45.5)	High risk 909 (54.5)	***P***-value
**Child’s characteristics**				
**Age (years)**	13.96 ± 2.89	13.50 ± 2.67	14.34 ± 2.86	< 0.001
**BMI (Kg/m**^**2**^)	19.43 ± 3.96	18.79 ± 3.75	19.96 ± 4.00	
**Sex**				0.32
Male	815(48.8)	361(47.5)	454(49.9)	
Female	854(51.2)	399(52.5)	455(50.1)	
**Paternal characteristics**				
**Age (years)**	47.20 ± 7.98	44.47 ± 6.71	49.46 ± 8.24	< 0.001
**Education**				< 0.001
Illiterate/ Primary	539.(32.3)	209(27.5)	330(36.3)	
Secondary	875(52.4)	424(55.8)	451(49.6)	
Higher	255(15.3)	127(16.7)	128(14.1)	
**Employment**				< 0.001
Housewife	236(14.1)	12(1.6)	224(24.6)	
Employed	1433(85.9)	748(98.4)	685(75.4)	
**Metabolic syndrome**				< 0.001
No	994(59.6)	760(100)	234(25.7)	
Yes	675(40.4)	00(0.0)	676(74.3)	
**Hypertension**				< 0.001
No	1333(79.9)	760(100)	573(63)	
Yes	336(20.1)	0(0.0)	336(37)	
**Bodyweight status**				< 0.001
Normal	630(37.7)	442(58.2)	188(20.7)	
Overweight	774(46.4)	310(40.8)	464(51.0)	
Obese	265(15.9)	8(1.1)	2.57(28.3)	
**Diabetes**				< 0.001
No	1493(89.5)	760(100)	733(80.6)	
Yes	176(10.5)	0(0.0)	176(19.4)	
**Smoking status**				< 0.001
No	1152(69.0)	473(62.2)	679(74.7)	
Yes	517(31.0)	227(37.8)	230(25.3)	
**Physical activity**				0.21
Low	1082(64.8)	496(65.3)	586(64.5)	
Moderate	255(15.3)	125(16.4)	130(14.3)	
High	332(19.9)	139(18.3)	193(21.2)	

Information on incidence rates by sex and parental cluster groups are presented in Table 3. Crude incidence rates (per 10,000 person-years) of HTN were 86 (95% CI: 71–106) and 38 (95% CI: 29–52) in boys and girls, respectively. Moreover, incidence rates (per 10,000 person-years) of HTN were 50 (95% CI: 40–63) and 80 (95% CI: 64–102) in the maternal low- and high-risk clusters, respectively. The incidence rates (per 10,000 person-years) of HTN in paternal low- and high-risk clusters were 53 (95% CI: 41–70) and 68 (95% CI: 56–84), respectively.

**Table 3:** Incidence rates (per 10,000 person-years) of hypertension, by sex and parental clusters

Variables	Incidence Rate	95% CI
**Sex**		
Male	86	71–106
Female	38	29–52
**Maternal risk clusters**		
Low-risk	50	40–63
High-risk	80	64–102
**Paternal risk clusters**		
Low risk	53	41–70
High risk	68	56–84
**Total**	61	52–73

**Fig. 3:**
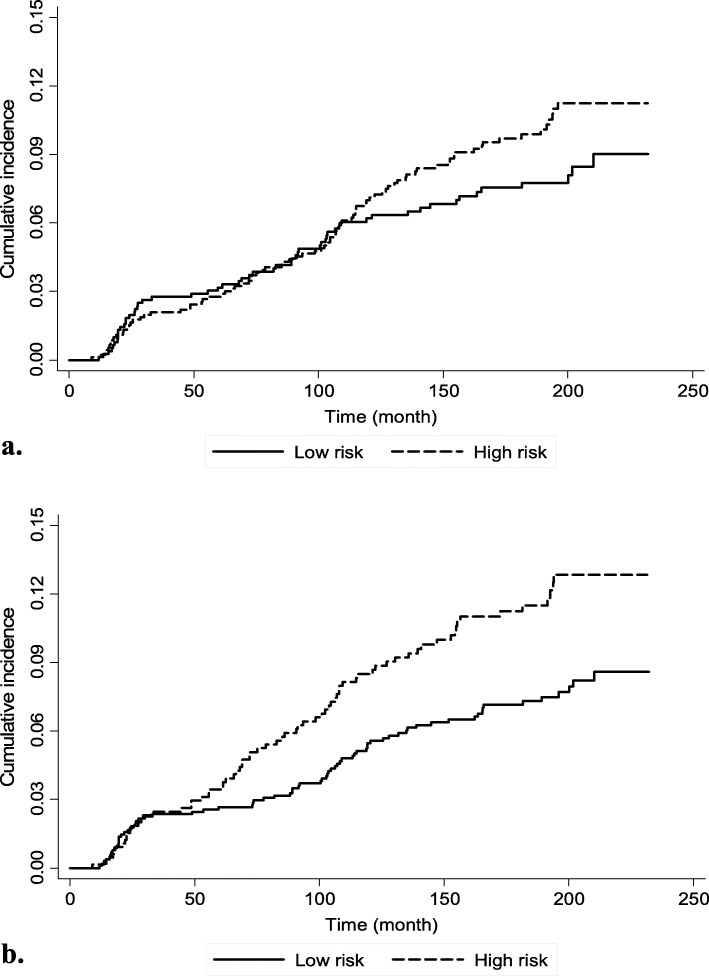
Kaplan-Meier cumulative incidence curve of hypertension in children according to different cluster groups related to **a** fathers’ and **b** mothers’ characteristics; the log-rank test *p*-value were 0.154 and 0.005, respectively

According to paternal and maternal risk clusters, Kaplan-Meier curves for incidence hypertention in children were presented respectively in Fig. 3a and b. The log-rank test showed a significant difference in the incidence of hypertension only for maternal risk clusters (*p* = 0.005). The association between parental risk clusters and incidence of hypertension in children using Cox proportional hazard models is presented in Table 4. Models 1 and 2 are unadjusted models for maternal and paternal risk clusters, respectively. Model 3 and 4 were models adjusted for child’s age, sex, and BMI. Model 5 is the final model that includes both parental risk clusters and is adjusted for child’s age, sex, and BMI. Findings indicate that only maternal clusters’ effect was significant on the incidence of HTN in children in both unadjusted (HR = 1.59, 95% CI: 1.15–2.21; *p* = 0.005) and adjusted models (HR = 1.46, 95% CI: 1.05–2.03; *p* = 0.025). In the final model, significant factors influencing the incidence of HTN in children were maternal cluster (HR = 1.44, 95% CI: 1.03–2.02; *p* = 0.03, Ref: low risk cluster), children’s sex (HR = 0.44, 95% CI: 0.31–0.62; *p* < 0.001, Ref: male) and children’s BMI (HR = 1.09, 95% CI: 1.05–1.13; *p* < 0.001).

**Table 4:** Hazard Ratios (HRs) and 95% confidence intervals for incidence of hypertension based on parental risk clusters

Models	Predictors	Risk clusters	HR (95% CI)	***P***-value
**Model 1**	Maternal cluster	Low risk (ref)	1	.
		High risk	1.59 (1.15–2.21)	0.005
**Model 2**	Paternal cluster	Low risk (ref)	1	.
		High risk	1.27 (0.91–1.78)	0.16
**Model 3**	Maternal cluster	Low risk (ref)	1	.
		High risk	1.46 (1.05–2.03)	0.025
	Sex	Male (ref)		.
		Female	0.44 (0.31–0.62)	< 0.001
	Age		0.97 (0.91–1.03)	0.30
	BMI		1.09 (1.05–1.14)	< 0.001
**Model 4**	Paternal cluster	Low risk (ref)	1	.
		High risk	1.14 (0.81–1.60)	0.44
	Sex	Male (ref)		.
		Female	0.44 (0.31–0.62)	< 0.001
	Age		0.97 (0.91–1.03)	0.34
	BMI		1.10 (1.06–1.14)	< 0.001
**Model 5**	Maternal cluster	Low risk (ref)	1	.
		High risk	1.44 (1.03–2.02)	0.03
	Paternal cluster	Low risk (ref)	1	.
		High risk	1.09 (0.77–1.53)	0.63
	Sex	Male (ref)	1	.
		Female	0.44 (0.31–0.62)	< 0.001
	Age		0.96 (0.90–1.03)	0.29
	BMI		1.09 (1.05–1.13)	< 0.001

The effects of child’s age, sex, and BMI were adjusted in multiple models (models 3, 4, and 5)

## Discussion

The current study investigated the association of maternal and paternal risk clusters with the incidence of HTN in school-aged children over a 13-year follow-up. Our results indicate cardio-metabolic risk factors as the most important variable distinguishing parental risk clusters in both parents. Furthermore, another finding was the significant prognostic values of maternal risk clusters to predict HTN in their children, which was not supported by our data regarding the paternal risk cluster. In addition, boys compared to girls and children with higher BMI were more likely to be hypertensive in the future.

In the current study regarding socio-demographic characteristics of parents, only employment status in fathers and age in both parents played important roles, a result consistent with previous data, suggesting the significant effect of family socioeconomic status (SES) defined as parental employment and family income on children’s general health and risk of CVD development in adulthood [34–36]. Accordingly, previous findings in Iran showed that moderate and low familial, economic status could increase the incidence of HTN in school-aged children [18]. The Young Finns Study tracking BP in children for 30 years until adulthood also indicated the contribution of parental occupation status in the elevation of offspring’s BP [37]. A potential explanation might be that parental unemployment and low income could result in mental and physical risk factors such as anxiety, depression, and obesity, resulting in increased BP in their children [35, 38].

According to current findings, in both parents, the most important factors in distinguishing high and low parental risk clusters were essentially cardio-metabolic risk factors, including MetS, HTN, and weight status, while physical activity was the least significant one. Most previous studies attempting to unveil the impact of parental cardio-metabolic risk factors in association with childhood HTN have considered these factors separately and underscored the importance of the family history of HTN and parental weight status in increasing risk of HTN in their children [7, 12, 39–41]. Even in Iran, HTN and obesity of parents were found to be potential risk factors resulting in elevated BP in children [17]. Most existing studies have not considered the synergic effects of the parental cardio-metabolic cluster on the BP level of their young offspring. However, in line with our findings, a cohort study in Japan revealed that clustering of maternal history of cardiometabolic disorders including HTN, diabetes, and dyslipidemia makes individuals more susceptible to clustering of these disorders in the future [42]. Meanwhile, another study in Germany referred to the interrelation of parental HTN, obesity, and smoking status as a combination of risk factors that could predispose children to HTN [15].

In the current study, only the maternal risk cluster appeared to have prognostic value in predicting HTN incidence in children, not the paternal one. The association between maternal HTN and weight status and children’s BP level has been documented previously [43]. However, limited data exist supporting the role of maternal cardio-metabolic characteristics as a risk cluster to predict offspring’s HTN; among those existing studies, a cohort study in Japan revealed that CVD outcomes including HTN in adult offspring were strongly influenced by the synergic effect of maternal cardio-metabolic history but not the paternal history of these disorders [42]. In Iran, the Isfahan Cohort Study also emphasizes the significance of screening maternal cerebrovascular diseases, including HTN, diabetes, hyperlipidemia, and obesity, in developing these risk factors in female children [44]. A combination of interrelated mechanisms, including environmental, genetic, and epigenetic factors, may elucidate the prognostic value of maternal cardiometabolic risk clusters in association with offspring’s HTN incidence [42]. Previous data suggest that healthy children of hypertensive mothers, compared to those without a family history of HTN, tend to have higher insulin levels which could explain the common pathway for developing CVD in those children [43]. Meanwhile, mitochondrial DNA-mediated inheritance could be a genetic explanation of maternal role in transmitting cardio-metabolic phenotypes including HTN to their children [45]. Greater shared environment with mothers resulting in similar lifestyles and behaviors, including dietary habits, may also contribute to these components [46].

In line with previous data, male children and those with high BMI in this study appeared to be at greater risk of developing HTN in the future. The predisposing role of gender in children to sustain high BP has been proposed in previous studies, implicating the higher incidence of HTN in male children [8, 10]. A systematic review on the prevalence of HTN in 122,053 adolescents, consistent with the current findings, also demonstrated that besides the methodological difference in studies, male adolescents have higher odds of high BP, especially in low- and middle-income countries [10]. Although most previous studies in Iran indicate higher BP incidence in boys [17, 47, 48], a few found only slightly significant differences or even contradictory results [18, 49]. The correlation between obesity and HTN in children is well established in literature which is in line with current findings [8, 50]. Studies from different parts of Iran have also documented the influential impact of high BMI on increasing BP in this age group [17, 18, 21, 48].

Among strengths of the current study, the large sample size, its longitudinal nature accompanied by long-term follow-up, and data collection for both parents are of significance. Some limitations of this study should also be mentioned. In this study, some parental factors, including uric acid levels, dietary intakes, and family income, were unavailable and were not included in the study analysis. Also, conducting the current study on an urban population could reduce the generalizability of the present findings, for which further research in suburban and rural areas is recommended.

In conclusion, the current results underscore the prognostic value of maternal risk cluster in predicting the incidence of HTN in their offspring. Highlighting this finding will be valuable for health policymakers to identify the most vulnerable children for HTN and designing preventive strategies.

## Data Availability

The datasets used and/or analyzed during the current study are available from the corresponding author on reasonable request.

## Abbreviations

BP: Blood pressure
BMI: Body mass index
CVD: Cardiovascular diseases
CI: Confidence interval
HRs: Hazard Ratios
HTN: Hypertension
MetS: Metabolic syndrome
SES: Socio-economic status
TLGS: Tehran Lipid and Glucose Study
WHO: World health organization
